# Effects of transcutaneous electrical nerve stimulation (TENS) on proinflammatory cytokines: protocol for systematic review

**DOI:** 10.1186/s13643-017-0532-5

**Published:** 2017-07-11

**Authors:** Tábata Cristina do Carmo Almeida, Francisco Winter dos Santos Figueiredo, Valter Cordeiro Barbosa Filho, Luiz Carlos de Abreu, Fernando Luiz Affonso Fonseca, Fernando Adami

**Affiliations:** 1 Faculdade de Medicina do ABC (FMABC), Laboratório de Epidemiologia e Análise de dados (ABC Medical School), Av. Lauro Gomes, 2000, Vila Sacadura Cabral, CEP: 09060-870 Santo André, SP Brazil; 2 Centre in Physical Activity and Health. Department of Physical Education, Federal University of Santa Catarina, Florianópolis, Brazil; 3 Faculdade de Medicina do ABC (FMABC), Laboratório de Delineamento de Estudos e Escrita Científica (ABC Medical School), Av. Lauro Gomes, 2000, Vila Sacadura Cabral, CEP: 09060-870 Santo André, SP Brazil; 4 Faculdade de Medicina do ABC (FMABC), Laboratório de Análises Clínicas (ABC Medical School), Av. Lauro Gomes, 2000, Vila Sacadura Cabral, CEP: 09060-870 Santo André, SP Brazil

**Keywords:** Transcutaneous electric nerve stimulation, TENS, Cytokines, Inflammation, Systematic review

## Abstract

**Background:**

Pain reduction can be achieved by lowering proinflammatory cytokine levels in the blood. Transcutaneous electrical nerve stimulation (TENS) is a non-invasive physiotherapeutic resource for pain management, but evidence on the effectiveness of this device at reducing proinflammatory cytokines in the blood is unclear. This study systematically reviews the literature on the effect of TENS on proinflammatory cytokines.

**Methods:**

A systematic review protocol was developed based on searches of articles in six electronic databases and references of retrieved articles, contact with authors, and repositories of clinical trials. Eligibility criteria: publication in peer-reviewed journals, randomized clinical trials, use of TENS in the experimental group, and pre- and post-measurements of proinflammatory cytokines in the blood. Selection of the studies and extraction of the data will be carried out by two reviewers independently. Characteristics of the study, participants, interventions and outcomes were extracted and described. Assessments were performed on the risk of bias, level of evidence and the size of the intervention effect in the studies, according to GRADE guidelines and the Cochrane Handbook for Systematic Reviews. Clinical and statistical assessments compared the effects of the interventions (meta-analysis), taking into consideration any influencing characteristics of the studies (e.g., methods and application sites).

**Discussion:**

We anticipate that this review will strengthen evidence-based knowledge of the effect of TENS on proinflammatory cytokines and, as a result, direct new studies to benefit patients with specific pathologies.

**Systematic review registration:**

PROSPERO, CRD42017060379.

**Electronic supplementary material:**

The online version of this article (doi:10.1186/s13643-017-0532-5) contains supplementary material, which is available to authorized users.

## Background

The first line of treatment for pain, according to international guidelines, is analgesic and anti-inflammatory medications. However, because of intolerable side effects in some individuals (e.g., gastritis, nausea and vomiting) [1] or ineffectiveness of these interventions, analgesic and non-pharmacological treatments with minimal side effects are needed [2]. Transcutaneous electrical nerve stimulation (TENS) has been increasingly studied as an alternative therapy.

To date, there has yet to be a definitive conclusion on the effectiveness of TENS in clinical practice. Some studies [1–5] show a reduction in pain intensity compared with control groups in a variety of disorders, indicating effective analgesia. However, there is great heterogeneity in clinical protocols (different combinations of TENS application parameters to treat different pathologies), small sample sizes, different methods of evaluation of the same outcomes or low methodological quality (e.g., lack of randomisation and blinding) [2, 4], which prevents the standardization of a clinical protocol.

TENS is a strategy that allows for interaction with the peripheral vascular system [6, 7] or with the autonomic nervous system [8, 9]. Studying its systemic effects may help determine the likely effectiveness of treatment when local access is limited or may shed light on other non-analgesic strategies, such as circulatory [6, 10], wound healing [7, 11], or inflammatory approaches [9, 12, 13].

Controlling inflammation in patients can reduce pain and improve their quality of life. In several clinical situations, such as in osteoarthritis [14], post-operative [15, 16], and even breast cancer [17, 18], proinflammatory cytokines are described as one of the main mediators of pain [18, 19].

When confronted with a stimulus, the body initiates a cascade of proinflammatory cytokines and induces the production and secretion of later or distal cytokines that perpetuate the inflammatory response. They cause sensitisation of nociceptors, which interact with the central nervous system to increase the perception of pain [19–21].

In the presence of pain, there are two possible mechanisms of action that may be involved in analgesia: (I) nociception blockade reduces the production of proinflammatory cytokines or (II) reduction of proinflammatory cytokines decreases pain intensity [15, 19, 22].

TENS acts on the afferent nerve fibers to block nerve transmission, an effect known as gating theory, or stimulates the release of opioids by the central nervous system [8]. These are both mechanisms that are known to decrease pain. In the context of this known interaction of pain and inflammation, this study sought to answer the following questions: (I) Can the blockage of nociception caused by TENS reduce proinflammatory cytokine levels? (II) If so, what are the application parameters of TENS required to change the proinflammatory cytokines?

To date, no literature review has been published focusing on the effect of TENS on the reduction of proinflammatory cytokines. This information would allow physiotherapists to apply TENS to persistent inflammatory processes that occur in various pathologies. To fill this gap in knowledge, we have developed a systematic review of studies of the effect of TENS on proinflammatory cytokine levels in adults. This protocol will consider the theoretical and methodological aspects of the published literature.

The aim of this systematic review protocol was to address the role of transcutaneous electrical nerve stimulation (TENS) on the reduction of blood levels of proinflammatory cytokines and also to determine how TENS parameters can affect cytokine levels.

## Methods

### Study design

This is a systematic review protocol for clinical trials prepared in accordance with the guidelines of the Preferred Reporting Items for Systematic Reviews and Meta-Analyses protocol (PRISMA-P) [23], as presented in Additional file 1.

### Study registration

This protocol was registered with the International Prospective Register of Systematic Reviews (PROSPERO) on 28 March 2017 (registration number: CRD 42017060379; http://www.crd.york.ac.uk/PROSPERO/).

### Identification of eligible studies

Included in this review will be studies published in peer-reviewed journals that meet the following eligibility criteria, organized by population, intervention, control, outcome, and study design (PICO framework) [24, 25].

#### Participants

Studies that address the application of TENS in adult humans (over 18 years of age) will be considered for the review. Animal studies will not be considered.

As TENS is used when symptoms occur (pain and/or inflammation), subjects with a variety of clinical conditions (e.g., chronic pain, acute post-operative pain, osteoarthritis, spine pain, and cancer pain) [5] will be included.

#### Intervention

TENS is a non-pharmacological analgesic therapy used in almost all medical specialties. The appliance generates an electrical current through cutaneous application interfaces, usually positioned around the area of pain. The application parameters are adjustable and determine the therapeutic effects of the current [5]. There are several modulations of TENS used in previous studies. We aim to discuss these modulations and their potential effects. The main application parameters of TENS [5, 8, 26–29] are described in Table 1.

**Table 1:** Parameters of TENS

Parameters	Characteristics
Frequency	High: from 80 to 150 Hertz (Hz) Low: from 1 to 20 Hertz (Hz)
Intensity	Low, moderate, high
Pulse width (duration)	50–250 micro seconds (μs)
Treatment time	Time in minutes (one session) Duration of treatment (days)
Modulation	Traditional TENS: high frequency (80–150 Hz), short pulses and intensity is often described as “Strong but comfortable”. (Pain gate activation) Acupunture TENS: lower frequency (1–5 Hz) with longer pulses (200–250 μs), and the intensity be greater than traditional TENS resulting in strong sensation. (opioid system activation)
Application (interface)	Non-invasive: electrodes Invasive: needles (electroacupunture)

Due to heterogeneity in TENS application, we chose not to make initial restrictions on the application parameters and only make adjustments in the analysis, according to the number of studies included with each possible intervention parameter. Furthermore, since TENS is generally used in symptomatic phase pathologies in which the medications cannot be stopped, it is better to consider the studies that use both medication and TENS concomitantly.

The TENS application parameters can trigger different mechanisms of action [5, 8]; and therefore, we defined specific control groups for each intervention group to somewhat homogenize the effects of the studies included.

The following interventions for the experimental group will be included:

1. TENS with application by electrodes;
2. TENS associated with pharmacological therapy (for pain and inflammation);
3. TENS with acupuncture needles (electro-acupuncture).

The application of TENS associated with other physiotherapeutic resources will not be included due to the difficulty of assigning causal relationships in interventions with multiple resources.

#### Types of study (control)

Randomized clinical trials should have an experimental group according to the interventions described above. Each intervention group should be paired with a specific control group. Only studies that fit these criteria (intervention X control) as described in Table 2 will be included.

**Table 2:** Relationship between intervention group and control group—required as inclusion criteria

Intervention group	Expected control group	Effect analyzed in the trial
TENS (Electrode application)	TENS placebo^a^ (Electrode application)	Effect of the TENS current on the reduction of proinflammatory cytokines
TENS (electrode) + pharmacological therapy^b^	TENS (electrode) placebo^a^ + pharmacological therapy^b^	
TENS (electrode) + pharmacological therapy^b^	Pharmacological Therapy^b^	
TENS by needles (Electro-acupuncture)	Acupuncture^c^	

^a^Placebo: the electrodes will be on the skin; there may be luminous indications that signal to the patient that the appliance is switched on, but the power is off

^b^Standard pharmacological therapy: prescription of the same drugs for both groups; usually analgesic or anti-inflammatory

^c^Acupuncture: needles should be on the same points as electro-acupuncture, but without the current working

#### Outcomes and prioritization

Only randomized clinical trials that have at least one measurement of at least one proinflammatory cytokine at the pre- and post-intervention stages of TENS will be considered for this review.

#### Primary outcomes

We will consider as the primary endpoint the level of any of the major proinflammatory cytokines [14, 18, 20, 30] described below:

1. Interleukin 1*β* (IL-1*β*);
2. Interleukin 1*α* (IL-1*α*);
3. Interleukin 2 (IL-2);
4. Tumor necrosis factor (TNF*α*);
5. Interleukin 6 (IL-6);
6. Interleukin 8 (IL-8).

#### Secondary outcomes

We will consider as a secondary outcome only pain assessed by the visual analogue scale (VAS) because of its relationship with inflammation [9, 12, 13].

### Search strategy

An electronic search of six databases will be carried out to identify publications that meet the selection criteria. The search will be carried out without limits to the date and language of the publication.

To identify the biochemical terms for the search, studies that addressed the subject of inflammatory cytokines will be consulted [20, 22, 31–34]. Keywords are selected according to Medical Subject Headings (MeSH) in the National Library of Medicine.

The organization of the terms for the search will be carried out according to the PICO strategy. To sensitize the search for articles, each database has described its strategy:

1. Medline (Pubmed) (Additional file 2): was used with MeSH terms and all their *entry terms*;
2. Scopus (Additional file 3);
3. Web of Science (Additional file 4);
4. Physiotherapy Evidence Database – PEDro (Additional file 5);
5. Cochrane Clinical Trials (Additional file 6);
6. EMBASE (Additional file 7).

In addition to the database searches, the following search strategies will be employed:

1. *Consultation of the reference lists of all original articles included*: After final selection, we will review references to identify clinical trials that may not have been found in the initial search.
2. *Contact with the authors*: (i) if complete articles are not available; (ii) after article selection, the authors will be consulted about other publications on the subject that may not have been found in the initial search; and (iii) if certain data are not available in the original article, such as data presented only in graphs.
3. *Searches in clinical trial repositories*: We will also consult the Clinical Trials (http://clinicaltrials.gov/) and the Brazilian Clinical Trials Registry (http://www.ensaiosclinicos.gov.br). For Clinical Trial﻿s, we will use the term “transcutaneous electrical nerve stimulation” for the search, and in the Brazilian registry, the term “*estimulação elétrica nervosa transcutânea*” is to be used. Eligibility criteria will be applied to original studies and those in the repositories for inclusion in this review. If we find eligible studies that are unpublished, we will contact the authors to obtain the results.

### Study records

#### Data management

The management of references and removal of duplicates will be performed with EndnoteX8, and articles will be managed using Excel spreadsheets and Review Manager 5 (RevMan) software (http://community.cochrane.org/tools/review-production-tools/revman-5).

#### Selection process

After searches in the proposed databases have been completed, a single library will be created in EndnoteX8. The duplicates will be removed with the help of the “find duplicate” tool. To ensure that all duplicates have been removed, we will check manually.

After removing duplicates, two identical libraries will be created in EndnoteX8 so that two reviewers can independently select articles. Also, two Excel spreadsheets will be created with references of all articles, using Endnote’s “copy formatted” tool in which the reasons for excluding articles will be justified.

There will be two levels to the selection process. At the first level, reviewers will screen articles by reading titles and abstracts in EndnoteX8 according to the following inclusion criteria: adult population, use of TENS as an intervention, dose of some proinflammatory cytokine, and randomized clinical trial. The reviewers must justify exclusion of any items in the spreadsheet.

Once the first level of selection is completed, the reviewers will hold a consensus meeting to evaluate the selection of articles to be evaluated at the second level. At this stage, if there is any divergence between the inclusion and exclusion criteria, a third reviewer will be consulted. At the end of this meeting, full article downloads will be performed, and as described earlier, we will create two libraries in EndnoteX8 and two Excel spreadsheets with identical contents for full post-read selection.

At the second level of selection, the reviewers will read the full texts. At this stage, the reviewers should evaluate the following inclusion criteria: the article must have at least one TENS intervention group and one control group according to Table 2 and present measurements of at least one proinflammatory cytokine before and after TENS. Exclusions must be justified in the spreadsheet.

After this selection, another consensus meeting will be held to review which articles will be considered eligible for review. If necessary, a third reviewer will also be used to resolve disagreements. At the end of this second level of the selection, a library will be created in EndnoteX8 with selected full articles.

Reference lists of included studies will be independently searched by one reviewer to identify additional studies.

#### Data items

For data extraction and management in spreadsheets in Excel, two reviewers will extract the data from the included articles; a third reviewer will resolve disagreements.

Reviewers will receive the spreadsheet (elaborated by TCCA) with all the variables to be filled. The main characteristics and variables that were used to build the spreadsheet are pre-established and described in Table 3.

**Table 3:** Characteristics and variables to be extracted from included articles

Characteristics	Variables
Population	Age in years; sex; primary diagnosis; medical specialty; pain history
Intervention (parameters of TENS)	Frequency; intensity, pulse duration, modality, application area, duration of treatment; application interface; time of follow-up
Biochemical parameters	Type of proinflammatory cytokines and evaluation of their expression in blood (level pre and post-intervention)
Methodological information	Sample size, primary endpoint studied (inflammation), other outcomes, randomization, blinding, patient eligibility criteria.

### Risk of bias and methodological quality

To reduce systematic biases and inferential error in the results of interest, we will evaluate the methodological quality based on evaluations of the strength of evidence and risk of bias, according to the Grades of Recommendation, Assessment, Development and Evaluation (GRADE) [35–41], and the guidelines of the Cochrane Handbook for Systematic Reviews [42].

The process for assessing methodological bias on individual studies will be performed in the RevMan program and the results will be presented risk of bias summary (review authors’ judgments about each risk of bias item for each included study).

The quality of final evidence, according to GRADE, will be classified as high, moderate, low, or very low. The study design is the starting point in assessing the quality of evidence. Randomized controlled trials are designated with the highest level of evidence because they are considered to be less prone to methodological limitations [35, 41]. There are five possible factors that subsequently diminish this quality: risk of bias [36], inconsistency [39], imprecision [38], indirect evidence [40], and publication bias [37].

The risk of bias (or methodological limitations) is assessed by the following factors: absence of allocation concealment (randomisation), absence of blinding, incomplete follow-up, selective reporting of outcomes, or other limitations (e.g., early termination of benefit study or use of outcome measures without validation) [36].

The quality of evidence is also reduced by indirect evidence, whereby participants, interventions, or outcomes evaluated in the study are substantially different from those considered in the research question (PICO) of the clinical trial [40].

Publication bias includes conflicts of interest or initial studies with positive results, especially with a small sample size, and the non-publication of negative results [37]. A funnel plot will be created to analyze the risk of publication bias in accepted studies, using the software Review Manager 5 (RevMan).

In the publication of the original article, if there are changes to the protocol, they will be detailed and justified in a specific section of the review.

### Data synthesis

We will present descriptive results relating to the study population, diagnoses, main treatment areas, objectives, and proinflammatory cytokine concentrations. Regarding the TENS application parameters, we intend to identify the most used values of frequency, intensity, pulse duration, treatment time, and modulation.

The size of the intervention effect will be calculated for each study using the mean difference with a 95% confidence interval (95% CI), according to equations used in the software Review Manager 5 (RevMan). The following parameters are required for this calculation: mean, standard deviation, and sample size of both the control and experimental group. When there is insufficient information to perform effect-size (ES) calculation, mean values and/or standard deviations will be requested from the authors of the original studies. In studies that have more than one experimental group besides TENS, the ES calculation will be performed independently for each group.

The results obtained in non-TENS experimental groups, or from studies that fail to meet the size criterium due to limited data, will be maintained in the systematic review, but not included in the meta-analysis if performed. The ES was classified according to the Cohen test [43] as very small (<0.20), small (0.20 to 0.49), intermediate (0.50 to 0.79) or large (≥0.80).

The decision of whether to include a study in a meta-analysis (for the mean difference in inflammatory markers using TENS application parameters) will be based on author consensus to avoid loss of clinical and statistical questions [42]. The following points will be considered:

i. Low level of clinical heterogeneity (ability to combine participants, intervention characteristics, and levels of proinflammatory cytokines);
ii. Low level of methodological heterogeneity (different methods used in the clinical trials are unlikely to influence the results).

Variability in the effects of the interventions will be tested for statistical heterogeneity using the chi-square test (*χ* ^2^) with the corresponding *p* value (Cochrane test) and through the *I* ^2^ statistic. The heterogeneity is considered low if *I* ^2^ ≤ 50% [44]. The level of significance will be 5%, and the analyses were performed in RevMan. For low heterogeneity, a fixed-effect meta-analysis will be used to estimate the treatment effect; for high heterogeneity, we will use the random effects model.

If the final consensus of the authors is not to perform the meta-analysis, we will present the results in a narrative synthesis.

#### Sensitivity and subgroup analyses (for the meta-analyses)

To evaluate the overall effect of an intervention, all experimental groups will be evaluated as a single category. Subgroup analyses may be performed, considering the clinical and statistical aspects, to identify factors acting as moderators of the intervention. This analysis will be performed to determine whether factors such as adequate sample size were attempted [42].

By identifying these factors, sensitivity analysis of the synthetic measurements will be performed. Although it is not possible to predict all of these adjustments in advance, we believe that factors related to different pain models (i.e., traumatic pain, neuropathic pain, etc.) can be used in the sensitivity analysis depending on the number of articles included.

The results will be presented in a forest plot, organized alphabetically according to the main author and in chronological order by year of publication.

To understand the relationship between TENS and proinflammatory cytokines, we will analyze variations in the levels of proinflammatory cytokines by considering characteristics of the intervention, such as frequency (high versus low), modulation (conventional versus acupuncture), and application interface (electrode versus needle), and the types of pathologies or medical areas.

## Preliminary report

**Fig. 1:**
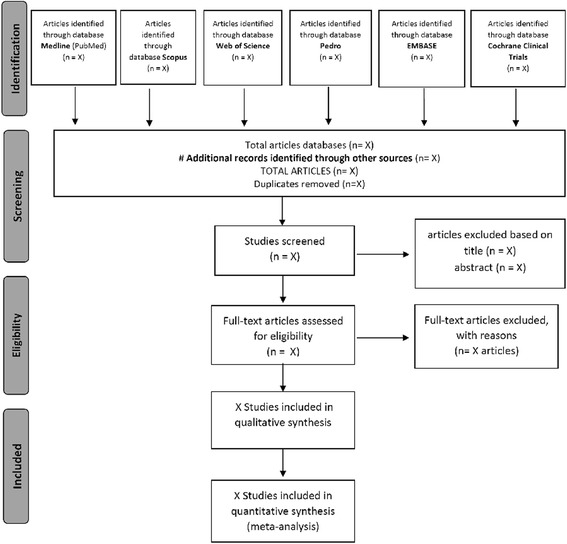
Research development process according to the PRISMA flow diagram

Figure 1 shows a flowchart of the research development process, according to PRISMA guidelines [45].

## Discussion

Although TENS is a widely-used method for pain management, some studies focus on the reduction of inflammation as an outcome. However, the mechanisms of action of TENS on the inflammatory process are not well understood. Despite scant evidence, the results of this review will direct further research into the effects of TENS treatment (in the case of poor evidence), or recommend interventions in case-specific studies (high quality of evidence).

A limitation of this review is that the studies may be influenced by short intervention times and/or small sample sizes. However, as TENS is performed in the symptomatic phase, participant inclusion is limited to specific pathologies and treatments performed in specific periods.

The TENS current can be adjusted by many application parameters (such as frequency, intensity, pulse size, and application interface) and used in various medical conditions. This adaptability makes it difficult to compare results when different methods are analyzed separately. However, regardless of the application parameter used, the grouping of interventions may represent the actual effect of the current on the outcome (judged here by inflammatory markers). TENS may reduce the inflammatory process and can be used as a physiotherapeutic treatment of pathologies marked by inflammation and pain.

## Additional files

Additional file 1: PRISMA-P (Preferred Reporting Items for Systematic review and Meta-Analysis Protocols) 2015 checklist: recommended items to address in a systematic review protocol. Completed PRISMA-P checklist outlining how each point has been addressed in the manuscript. (PDF 287 kb)

Additional file 2: Search strategy from Medline database. Description of the search terms according to the Medline (Pubmed) database. (PDF 186 kb)

Additional file 3: Search strategy from Scopus database. Description of the search terms according to the Scopus database. (PDF 167 kb)

Additional file 4: Search strategy from Web of Science database. Description of the search terms according to the Web of Science database. (PDF 291 kb)

Additional file 5: Search strategy from Physiotherapy Evidence Database - *PEDro* database. Description of the search terms according to the *PEDro* database. (PDF 176 kb)

Additional file 6: Search strategy from Cochrane Clinical Trials database. Description of the search terms according to the Cochrane Clinical Trials database. (PDF 290 kb)

Additional file 7: Search strategy from EMBASE database. Description of the search terms according to the EMBASE database. (PDF 183 kb)

## Abbreviations

CI: Confidence interval
ES: Effect-size
GRADE: Grades of Recommendation, Assessment, Development and Evaluation
IL-1*α*: Interleukin 1*α*
IL-1*β*: Interleukin 1*β*
IL-2: Interleukin 2
IL-6: Interleukin 6
IL-8: Interleukin 8
MeSH: Medical Subject Headings
PEDro: Physiotherapy Evidence Database
PICO strategy: Population, intervention, control, outcomes
PRISMA: Preferred reporting items for systematic reviews and meta-analyses
PRISMA-P: Preferred reporting items for systematic review and meta-analysis protocols
PROSPERO: International Prospective Register of Systematic Reviews
RevMan: Review Manager software
TENS: Transcutaneous electrical nerve stimulation
TNF*α*: Tumor necrosis factor alpha
VAS: Visual analogue scale
χ^2^: Chi-square test
