# Supplementary material for: Effects of transcutaneous electrical nerve stimulation (TENS) on proinflammatory cytokines: protocol for systematic review
Source: Syst Rev. 2017 Jul 11;6:139. doi: 10.1186/s13643-017-0532-5 (PMC5505047; doi:10.1186/s13643-017-0532-5)
Supplement: Supplementary file 2 — Search strategy from Medline database. Description of the search terms according to the Medline (Pubmed) database. (PDF 186 kb) [file 13643_2017_532_MOESM2_ESM.pdf]

Additional file 2: Search strategy from Medline database

	Database: Medline<br>**Descriptors**
**#1**	adult **OR** adults **OR** human **OR** humans
**#2**	Transcutaneuos Electric Nerve Stimulation + *Entry Terms* (MeSH)<br>Transcutaneous Electric Nerve Stimulation **OR** Electrical Stimulation, Transcutaneous **OR** Stimulation, Transcutaneuos Electrical **OR** Transcutaneous Electrical Stimulation **OR** Percutaneuos Electric Nerve Stimulation **OR** Transdermal Electrostimulation **OR** Electrostimulation, Transdermal **OR** TENS **OR** Transcutaneous Electrical Nerve Stimulation **OR** Transcutaneous Nerve Stimulation **OR** Nerve Stimulation, Transcutaneuos **OR** Stimulation, Transcutaneous Nerve **OR** Electric Stimulation, Transcutaneous **OR** Stimulation, Transcutaneous Electric **OR** Transcutaneous Electric Stimulation **OR** Percutaneuos Electrical Nerve Stimulation **OR** Analgesic Cutaneous Electrostimulation **OR** Cutaneous Electrostimulation, Analgesic **OR** Electrostimulation, Analgesic Cutaneous **OR** Electroanalgesia
**#3**	“randomized controlled trial” [Publication Type] **OR** “controlled clinical trial” [Publication Type] **OR** “randomized controlled trials” [MeSH Terms] **OR** “random allocation” [MeSH Terms] **OR** “double blind method” [MeSH Terms] OR single blind method [MeSH Terms] **OR** “clinical trial” [Publication Type] OR “clinical trials” [MeSH Terms] **OR** single* [Text Word] **OR** double* [Text Word] **OR** treble* [Text Word] **OR** triple* [Text Word] **OR** placebos [MeSH Terms] **OR** placebo* [Text Word] **OR** random* [Text Word] **OR** “research design” [MeSH Terms] **OR** “comparative study” [MeSH Terms] **OR** “evaluation studies” [MeSH Terms] **OR** follow-up stud* [MeSH Terms] **OR** prospective stud* [MeSH Terms] **OR** control* [Text Word] **OR** prospectiv* [Text Word] **OR** volunteer* [Text Word] **AND NOT** "animals"[MeSH Terms] **AND NOT** ("humans"[MeSH Terms] **AND** "animals"[MeSH Terms])
**#4**	Chemokines + Entry Terms (MeSH)<br>Chemokines **OR** Cytokines, Chemotactic **OR** Intercrines **OR** Chemotactic Cytokines **OR** cytokines **OR** cytokine.
**#5**	**#1** AND **#2** AND **#3** AND **#4**
**#6**	**Limits**: article types (Clinical Trial); Species (humans); age group (19 + years); without limitation of language or year of publication.
