# Supplementary material for: Effects of transcutaneous electrical nerve stimulation (TENS) on proinflammatory cytokines: protocol for systematic review
Source: Syst Rev. 2017 Jul 11;6:139. doi: 10.1186/s13643-017-0532-5 (PMC5505047; doi:10.1186/s13643-017-0532-5)
Supplement: Supplementary file 3 — Search strategy from Scopus database. Description of the search terms according to the Scopus database. (PDF 167 kb) [file 13643_2017_532_MOESM3_ESM.pdf]

Additional file 3: Search strategy from Scopus database.

	Database: Scopus<br>**Descriptors**
**#1**	TITLE-ABS-KEY (adult **OR** adults **OR** human **OR** humans)
**#2**	TITLE-ABS-KEY (Transcutaneous Electric Nerve Stimulation **OR** Electrical Stimulation, Transcutaneous **OR** Stimulation, Transcutaneuos Electrical **OR** Transcutaneous Electrical Stimulation **OR** Percutaneuos Electric Nerve Stimulation **OR** Transdermal Electrostimulation **OR** Electrostimulation, Transdermal **OR** TENS **OR** Transcutaneous Electrical Nerve Stimulation **OR** Transcutaneous Nerve Stimulation **OR** Nerve Stimulation, Transcutaneuos **OR** Stimulation, Transcutaneous Nerve **OR** Electric Stimulation, Transcutaneous **OR** Stimulation, Transcutaneous Electric **OR** Transcutaneous Electric Stimulation **OR** Percutaneuos Electrical Nerve Stimulation **OR** Analgesic Cutaneous Electrostimulation **OR** Cutaneous Electrostimulation, Analgesic **OR** Electrostimulation, Analgesic Cutaneous **OR** Electroanalgesia)
**#3**	TITLE-ABS-KEY ("randomized controlled trial" **OR** "controlled clinical trial" **OR** "randomized controlled trials" **OR** "random allocation" **OR** "double blind method" **OR** "single blind method" **OR** "clinical trial" **OR** "clinical trials") **OR** TITLE-ABS-KEY (clinical* AND trial*) OR TITLE-ABS-KEY (single* OR double* **OR** treble* **OR** triple* **OR** placebos **OR** placebo* **OR** random* **OR** "research design" **OR** "comparative study" **OR** "evaluation studies" **OR** follow-up stud* **OR** prospective stud* **OR** control* **OR** prospectiv* **OR** volunteer*) **AND NOT** TITLE-ABS-KEY (animal) **AND NOT** TITLE-ABS-KEY (human **AND** animal)
**#4**	TITLE-ABS-KEY (Chemokines **OR** Cytokines, Chemotactic **OR** Intercrines **OR** Chemotactic Cytokines **OR** Cytokines **OR** Cytokine)
**#5**	*SEARCH (COMBINE QUERIES)*<br>**#1** AND **#2** AND **#3** AND **#4**
**#6**	**Limits:** document type (article and article in press); without limitation of language or year of publication.
