# Supplementary material for: Effects of transcutaneous electrical nerve stimulation (TENS) on proinflammatory cytokines: protocol for systematic review
Source: Syst Rev. 2017 Jul 11;6:139. doi: 10.1186/s13643-017-0532-5 (PMC5505047; doi:10.1186/s13643-017-0532-5)
Supplement: Supplementary file 4 — Search strategy from Web of Science database. Description of the search terms according to the Web of Science database. (PDF 291 kb) [file 13643_2017_532_MOESM4_ESM.pdf]

Additional file 4: Search strategy from Web of Science database.

	Database: Web of Science<br>**Descriptors**
**#1**	TS= (adult) **OR** TS=(adults) OR TS=(human) **OR** TS=(humans)
**#2**	TS=(“Transcutaneous Electric Nerve Stimulation”) **OR** TS=(“Electrical Stimulation, Transcutaneous”) **OR** TS=(“Stimulation, Transcutaneuos Electrical”) **OR** TS=(“Transcutaneous Electrical Stimulation”) **OR** TS=(“Percutaneuos Electric Nerve Stimulation”) **OR** TS=(“Transdermal Electrostimulation”) **OR** TS=(“Electrostimulation, Transdermal”) **OR** TS=(TENS) **OR** TS=(“Transcutaneous Electrical Nerve Stimulation”) **OR** TS=(“Transcutaneous Nerve Stimulation”) **OR** TS=(“Nerve Stimulation, Transcutaneuos”) **OR** TS=(“Stimulation, Transcutaneous Nerve”) **OR** TS=(“Electric Stimulation, Transcutaneous”) **OR** TS=(“Stimulation, Transcutaneous Electric”) **OR** TS=(“Transcutaneous Electric Stimulation”) **OR** TS=(“Percutaneuos Electrical Nerve Stimulation”) **OR** TS=(“Analgesic Cutaneous Electrostimulation”) **OR** TS=(“Cutaneous Electrostimulation, Analgesic”) **OR** TS=(“Electrostimulation, Analgesic Cutaneous”) **OR** TS=(Electroanalgesia)
**#3**	TS=(“randomized controlled trial”) **OR** TS=(“controlled clinical trial”) **OR** TS=(“randomized controlled trials”) **OR** TS=(“random allocation”) **OR** TS=(“double blind method”) **OR** TS=(“single blind method”) **OR** TS=(“clinical trial”) **OR** TS=(“clinical trials”) **OR** (TS=(clinical*) **AND** TS=(trial*)) **OR** TS=(single) **OR** TS=(double) **OR** TS=(treble*) **OR** TS=(triple*) **OR** TS=(placebos) **OR** TS=(placebo*) **OR** TS=(random*) **OR** TS=(“research design”) **OR** TS=(“comparative study”) **OR** TS=(“evaluation studies”) **OR** TS=(follow-up stud*) **OR** TS=(prospective stud*) **OR** TS=(control*) **OR** TS=(prospectiv*) **OR** TS=(volunteer*) **NOT** TS=(animal) **NOT** TS=(human **AND** animal)
**#4**	TS= (Chemokines) **OR** TS= (“Cytokines, Chemotactic”) **OR** TS=(Intercrines) **OR** TS=(“Chemotactic Cytokines”) **OR** TS=(Cytokines) **OR** TS=(Cytokine)
**#5**	Search History (combine)<br>**#1** AND **#2** AND **#3** AND **#4**
**#6**	**Limits:** documents types (articles); without limitation of language or year of publication.
