# Supplementary material for: Effects of transcutaneous electrical nerve stimulation (TENS) on proinflammatory cytokines: protocol for systematic review
Source: Syst Rev. 2017 Jul 11;6:139. doi: 10.1186/s13643-017-0532-5 (PMC5505047; doi:10.1186/s13643-017-0532-5)
Supplement: Supplementary file 5 — Search strategy from Physiotherapy Evidence Database - PEDro database. Description of the search terms according to the PEDro database. (PDF 176 kb) [file 13643_2017_532_MOESM5_ESM.pdf]

Additional file 5: Search strategy from Physiotherapy Evidence Database – *PEDro*

	Database: Physiotherapy Evidence Database – PEDro<br><br>**Descriptors**
**#1**	Choose the option # Advanced search #
**#2**	Abstract & Title: *Transcutaneous electrical nerve stimulation*
**#3**	Therapy: select *“electrotherapies, heat, cold”*
**#4**	Problem: *not select anything*
**#5**	Body Part: *not select anything*
**#6**	Subdiscipline: *not select anything*
**#7**	Topic: select *“no appropiate value in this field”*
**#8**	Method: select *“clinical trial”*
**#9**	# When searching: (x) match all search terms (AND)#
