# Supplementary material for: Effects of transcutaneous electrical nerve stimulation (TENS) on proinflammatory cytokines: protocol for systematic review
Source: Syst Rev. 2017 Jul 11;6:139. doi: 10.1186/s13643-017-0532-5 (PMC5505047; doi:10.1186/s13643-017-0532-5)
Supplement: Supplementary file 6 — Search strategy from Cochrane Clinical Trials database. Description of the search terms according to the Cochrane Clinical Trials database. (PDF 290 kb) [file 13643_2017_532_MOESM6_ESM.pdf]

Additional file 6: Search strategy from Cochrane Clinical Trials database.

	Database: Cochrane Clinical Trials<br>**Descriptors**
**#1**	(adult):ti,ab,kw **OR** (adults):ti,ab,kw **OR (**human):ti,ab,kw **OR** (humans):ti,ab,kw
**#2**	(“Transcutaneous Electric Nerve Stimulation”):ti,ab,kw **OR** (“Electrical Stimulation, Transcutaneous”):ti,ab,kw **OR** (“Stimulation, Transcutaneuos Electrical”):ti,ab,kw **OR** (“Transcutaneous Electrical Stimulation”):ti,ab,kw **OR** (“Percutaneuos Electric Nerve Stimulation”):ti,ab,kw **OR** (“Transdermal Electrostimulation”):ti,ab,kw **OR** (“Electrostimulation, Transdermal”):ti,ab,kw **OR** (TENS):ti,ab,kw **OR** (“Transcutaneous Electrical Nerve Stimulation”):ti,ab,kw **OR (“**Transcutaneous Nerve Stimulation”):ti,ab,kw **OR** (“Nerve Stimulation, Transcutaneuos”):ti,ab,kw **OR** (“Stimulation, Transcutaneous Nerve”):ti,ab,kw **OR** (“Electric Stimulation, Transcutaneous”):ti,ab,kw **OR** (“Stimulation, Transcutaneous Electric”):ti,ab,kw **OR** (“Transcutaneous Electric Stimulation”):ti,ab,kw **OR** (“Percutaneuos Electrical Nerve Stimulation”):ti,ab,kw **OR** (“Analgesic Cutaneous Electrostimulation”):ti,ab,kw **OR** (“Cutaneous Electrostimulation, Analgesic”):ti,ab,kw **OR** (“Electrostimulation, Analgesic Cutaneous”):ti,ab,kw **OR** (Electroanalgesia):ti,ab,kw
**#3**	(randomized controlled trial):pt OR (controlled clinical trial):pt OR me (randomized controlled trials) OR me (random allocation) OR me (double blind method) OR me (single blind method) OR (clinical trial):pt OR me (clinical trials) OR (clinical* AND trial*):ti,ab,kw OR (single*):ti,ab,kw OR (double*):ti,ab,kw OR (treble*):ti,ab,kw OR (triple*):ti,ab,kw OR me (placebos) OR (placebo*):ti,ab,kw OR (random*):ti,ab,kw OR (“research design”):ti,ab,kw OR me (comparative study) OR me (evaluation studies) OR me (follow-up stud*) OR me (prospective stud*) OR (control*):ti,ab,kw OR (prospectiv*):ti,ab,kw OR (volunteer*):ti,ab,kw AND NOT (animal):ti,ab,kw AND NOT (human AND animal):ti,ab,kw
**#4**	(Chemokines):ti,ab,kw **OR** (“Cytokines, Chemotactic”):ti,ab,kw **OR (**Intercrines):ti,ab,kw **OR** (“Chemotactic Cytokines”):ti,ab,kw **OR** (cytokines):ti,ab,kw **OR** (cytokine):ti,ab,kw
**#5**	**#1** AND **#2** AND **#3** AND **#4**
**#6**	**Limits:** trials; without limitation of language or year of publication.
