# Supplementary material for: Effects of transcutaneous electrical nerve stimulation (TENS) on proinflammatory cytokines: protocol for systematic review
Source: Syst Rev. 2017 Jul 11;6:139. doi: 10.1186/s13643-017-0532-5 (PMC5505047; doi:10.1186/s13643-017-0532-5)
Supplement: Supplementary file 7 — Search strategy from EMBASE database. Description of the search terms according to the EMBASE database. (PDF 183 kb) [file 13643_2017_532_MOESM7_ESM.pdf]

Additional file 7: Search strategy from EMBASE database.

	Database: EMBASE<br>**Descriptors**
**#1**	adult **OR** adults **OR** human **OR** humans
**#2**	“Transcutaneous Electric Nerve Stimulation” **OR** “Electrical Stimulation, Transcutaneous” **OR** “Stimulation, Transcutaneuos Electrical” **OR** “Transcutaneous Electrical Stimulation” **OR** “Percutaneuos Electric Nerve Stimulation” **OR** “Transdermal Electrostimulation” **OR** “Electrostimulation, Transdermal” **OR** TENS **OR** “Transcutaneous Electrical Nerve Stimulation” **OR** “Transcutaneous Nerve Stimulation” **OR** “Nerve Stimulation, Transcutaneuos” **OR** “Stimulation, Transcutaneous Nerve” **OR** “Electric Stimulation, Transcutaneous” **OR** “Stimulation, Transcutaneous Electric” **OR** “Transcutaneous Electric Stimulation” **OR** “Percutaneuos Electrical Nerve Stimulation” **OR** “Analgesic Cutaneous Electrostimulation” **OR** “Cutaneous Electrostimulation, Analgesic” **OR** “Electrostimulation, Analgesic Cutaneous” **OR** Electroanalgesia
**#3**	“randomized controlled trial” OR “controlled clinical trial” OR “randomized controlled trials”/exp OR “random allocation”/exp OR “double blind method”/exp OR “single blind method”/exp OR “clinical trial” OR “clinical trials”/exp OR (clinical* AND trial*) OR single* OR double* OR treble* OR triple* OR placebos/exp OR placebo* OR random* OR “research design”/exp OR “comparative study”/exp OR “evaluation studies”/exp OR (follow-up stud*/exp) OR (prospective stud*/exp) OR control* OR prospectiv* OR volunteer* NOT animal NOT (human AND animal)
**#4**	Chemokines **OR** “Cytokines, Chemotactic” **OR** Intercrines **OR** “Chemotactic Cytokines” **OR** cytokines **OR** cytokine.
**#5**	**#1** AND **#2** AND **#3** AND **#4**
**#6**	**Limits:** humans; without limitation of language or year of publication.
